# The Effect of Dietary Fiber Compositions on the Therapeutic Outcome of Combined Radio‐ and Immunotherapy in a Preclinical Cancer Model

**DOI:** 10.1002/mnfr.70370

**Published:** 2026-01-20

**Authors:** Annemarie J. F. Westheim, Ludwig J. Dubois, Elia Prades‐Sagarra, Jiyang Chan, Alexander M. A. van der Wiel, Natasja G. Lieuwes, Rianne Biemans, Ying Cong, Tom Houben, Dennis M. Meesters, Ala Yaromina, Miriam van Dijk, Jeroen van Bergenhenegouwen, Ardy van Helvoort, Ramon C. J. Langen, John Penders, Ronit Shiri‐Sverdlov, Jan Theys

**Affiliations:** ^1^ The M‐Lab, Department of Precision Medicine, GROW‐Research Institute for Oncology and Reproduction Maastricht University Maastricht the Netherlands; ^2^ Department of Genetics and Cell Biology, NUTRIM‐Research Institute of Nutrition and Translational Research in Metabolism Maastricht University Maastricht the Netherlands; ^3^ Department of Medical Microbiology, Infectious Diseases and Infection Prevention, NUTRIM‐Research Institute of Nutrition and Translational Research in Metabolism Maastricht University Maastricht the Netherlands; ^4^ Danone Nutricia Research Utrecht the Netherlands; ^5^ Department of Respiratory Medicine, NUTRIM‐Research Institute of Nutrition and Translational Research in Metabolism Maastricht University Medical Centre Maastricht the Netherlands

**Keywords:** cancer, diet, fiber, immunotherapy, radiotherapy

## Abstract

Several studies demonstrated curative responses of combined radio‐immunotherapy, although not standard‐of‐care for most cancers. Increased fiber intake has been associated with improved radiotherapy and immunotherapy outcomes, but fiber compositions’ impact remains unclear. This study aimed to explore whether dietary fiber composition influences the therapeutic outcome of combined radio‐immunotherapy in a preclinical cancer model. A syngeneic mouse model of colon cancer (CT26) (female BALB/cOla Hsd) was used. Mice were randomized into three groups (*n* = 12) of iso‐caloric diets with different fiber compositions. Five instances of local radiotherapy on tumors, combined with injections of anti‐PD‐L1, were administered over 5 and 10 days. Diets’ impact was assessed on progression‐free survival, SCFA levels in fecal and cecal samples, gut microbiome composition, and immunological profile. Progression‐free survival was different between compositions, as well as their gut microbiota community structure, at all measured time‐points. Therapeutic outcome (cure) was negatively associated with the relative abundance of *Bacteroides* and positively with *Atopobiaceae Family*. There was no association with SCFA levels. Cured mice displayed smaller spleens containing increased proportions of CD8+ T‐cells and decreased proportions of myeloid‐derived suppressor cells. Our data suggest that fiber composition may influence therapeutic outcome of combined radio‐immunotherapy treatment in vivo.

## Graphical abstract

To study whether dietary fiber composition influences the therapeutic outcome of combined radio‐immunotherapy, mice were subcutaneously injected with syngeneic CT26 colon cancer cells and randomized into three groups of iso‐caloric diets with different fiber compositions. Animals were treated with local radiotherapy on tumors, combined with intraperitoneal injections of anti‐PD‐L1. Diets’ impact was assessed on progression‐free survival. Our data suggest that fiber composition may influences therapeutic outcome of combined radio‐immunotherapy treatment in vivo.

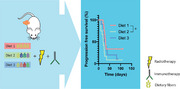

**Abbreviations**
AIN: American Institute of Nutrition
ASV: amplicon sequence variant
CT: Colon Tumor
FBS: fetal bovine serum
HFD: high‐fat diet
ICI: immune checkpoint inhibitors
IT: immunotherapy
LinDA: linear models for differential abundance analysis
MDSC: myeloid‐derived suppressor cells
*n*
: number
NK: natural killer cells
PFS: progression‐free survival
RT: radiotherapy
T4Xsv: four times the initial starting volume

## 1 Introduction

The World Health Organization has highlighted cancer as the second highest contributor to global mortality, with an estimate of 10 million deaths attributed to cancer in 2020, and this trend continues to rise [1, 2]. Common anti‐cancer therapies include surgery, chemotherapy, radiotherapy (RT), and immunotherapy (IT). RT has been shown to trigger immunogenic cell death, making it particularly effective when combined with IT [3, 4, 5]. Although combined RT/IT approaches are not yet standard‐of‐care for most cancer types, (pre)clinical studies already demonstrated long‐lasting curative responses [3, 6, 7, 8].

Recently, there has been increased interest in how the gut microbiota can promote or prevent cancer formation [9]. In addition, several studies report that effectiveness of anti‐cancer therapies are influenced by the gut microbiota composition [10, 11, 12, 13]. The gut microbiome influences IT efficacy by regulating the immune system, via modulating both innate and adaptive immunity through its metabolites [14]. Moreover, gut microbiome dysbiosis has been implicated in cancer cachexia through pathways of systemic inflammation and muscle wasting [15], which has been described to be associated with diminished responses to IT [16].

A key determinant shaping the gut microbiome composition is dietary fiber intake. Dietary fibers can be categorized based on source, chemical structure, solubility, or their capacity to be fermented by gut microbiota [17]. Preclinical data have demonstrated that microbiome changes, induced by fiber‐rich diets, were associated with sensitization of tumor xenografts to irradiation [18]. SCFAs, a specific group of metabolites produced upon microbiota fiber‐fermentation, are suggested to enhance sensitivity to irradiation [18, 19]. Epidemiological and preclinical studies also have described positive associations between SCFAs and IT efficacy [20, 21, 22]. Moreover, mice fed fiber‐rich diets demonstrated enhanced anti‐tumor responses to immune checkpoint inhibitors (ICI), with delayed tumor outgrowth compared to those on fiber‐poor diets [20, 21, 22]. Furthermore, Spencer et al. demonstrated a positive correlation between dietary fiber consumption and improved responses to ICIs in humans [23]. However, despite evidence that increased fiber intake can improve RT and IT efficacy, it is not yet known whether dietary fiber composition influences combined RT/IT outcome. To investigate this, three fiber mixes containing equal total fiber contents, but differing in fiber composition were designed. In the current study, we assessed the impact of these three fiber compositions on nutritional status and outcome of RT/IT combination therapy. Additionally, we analyzed gut microbiome diversity, composition and metabolites in the three groups, as well as the immunological profiles in the spleens.

## 2 Materials and Methods

### 2.1 Cell Lines

Colon tumor (CT) 26 cells (Philogen S.p.A., Siena) were cultured in DMEM (Sigma‐Aldrich, Zwijndrecht) supplemented with 10% fetal bovine serum (FBS) is a humidified 5% CO_2_ chamber at 37°C.

### 2.2 Animals

#### 2.2.1 Ethical Statement

All animal experiments adhered to the animal welfare guidelines established by the local institution. Experiments received approval from the Animal Ethical Committee of the University of Maastricht, the Netherlands (ADV10700202114406). Procedures followed were strictly guided by the principles outlined in the Declaration of Helsinki of 1975, as amended in 2000. The Code of Practice animal experiments in cancer research, as devised by the Working Party on Animal Experimentation in Cancer Research, was followed.

#### 2.2.2 Dosage Regimen

The semi‐synthetic American Institute of Nutrition (AIN)‐93 M diet (SNIFF, Soest), which contains 47.5 g/kg cellulose as a source of fiber, was used as a base (composition 1). Cellulose (non‐digestible/non‐fermentable) is commonly used in semi‐synthetic rodent diets. To investigate effects of fiber composition on RT/IT efficacy, modified versions of the AIN93M diet were designed in which cellulose was substituted with arabinoxylans, beta‐glucans, pectin, starch, and prebiotic oligosaccharides. Three fiber compositions each containing equal total fiber contents, while differing in fiber composition (Table S1), were tested in a subcutaneous CT26 mouse model treated with combined RT/IT. Mice were offered respective diets in pellet form ad libitum. Food intake was assessed on a weekly basis by weighing the uneaten food. After 1 week, fresh pellets were added, and on a bi‐weekly basis food was completely replenished.

#### 2.2.3 Experimental Design

**FIGURE 1:**
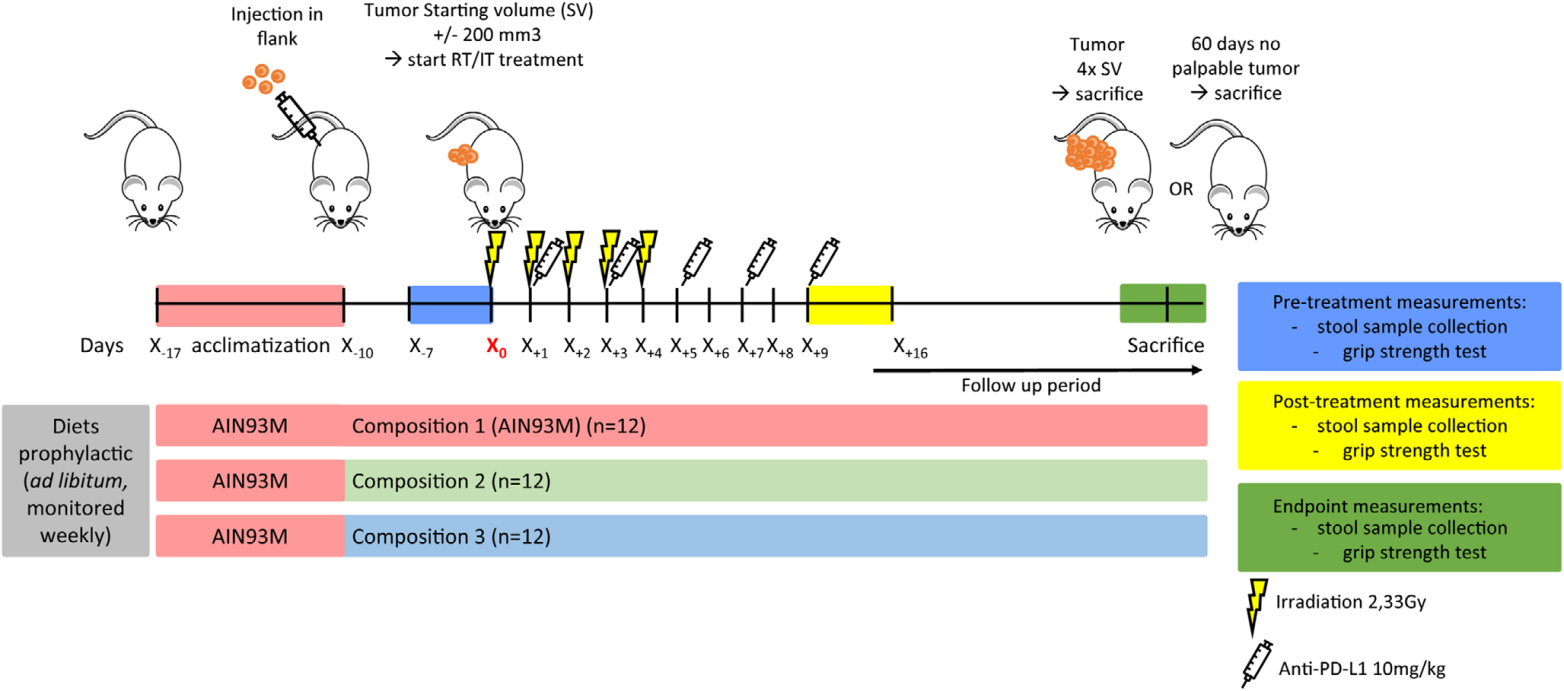
Schematic overview of the in vivo experiment.

Eight‐ to twelve‐week‐old immunocompetent female BALB/cOla Hsd mice (Envigo, Horst) were socially housed (*n* = 4), at room temperature (21 ± 1°C), following a 12:12 dark‐light cycle, in GM500 IVC cages (Tecniplast, Buguggiate) containing Corn cob bedding (JRS Lignocel, Rosenberg), PVC‐shelter, and nesting material. Food and drinking water (autoclaved, softened, acidified (pH = 2.5)) were provided ad libitum. Following a 1‐week acclimatization with AIN93M food pellets, mice were randomly allocated (at the cage level) to different groups (*n* = 12 per group), each receiving an iso‐caloric diet based on AIN93M (schematic timeline shown in Figure 1). Animal body weights were monitored 2‐3 times per week and not corrected for tumor mass. Researchers remained blinded to the diets, with blinding only unveiled at the conclusion of the study. To induce tumors, 1.5 × 10^6^ syngeneic CT26 cells were mixed with basement membrane matrix (MatrigelTM, BD Biosciences, San Diego) and injected subcutaneous into the right flank of each mouse. Throughout the study, tumor dimensions were measured with a Vernier caliper and tumor volume was calculated using the formula “(π/6) × length × width × height,” with corrections made for skin thickness (0.5 mm). Upon reaching a starting volume of 228.7 ± 57.6 mm^3^, tumors underwent local irradiation with 2.33 Gy (Varian Truebeam linear accelerator, 15 MeV [24]) administered daily for 5 consecutive days. Concurrently, mice were intraperitoneally injected (10 mg/kg) with a rat anti‐murine PD‐L1 antibody (Bioxcell, clone 10F.9G2, Huissen) diluted in sterile saline, starting 1 day after the first irradiation session and repeated every alternate day for a total of five injections. Experimental endpoint was predefined as reaching a tumor volume four times the initial starting volume (t4xSV), or observing no palpable tumor for 60 consecutive days (“cured”). Medium progression‐free survival (PFS) was defined in survival curves as the point where curves reach 50% survival. Animal welfare was monitored daily and predefined humane endpoints were applied under supervision of the local veterinarian whenever necessary. Humane endpoints were applied for two mice that dropped‐out prematurely, one due to intraperitoneal tumor growth and the other due to model‐unrelated clinical signs of neurological pathology.

#### 2.2.4 Longitudinal Measurements

Prior to anti‐cancer treatment with RT/IT, 1‐day post‐RT/IT, and at sacrifice, fecal samples were collected in screw cap vials, snap‐frozen in liquid nitrogen, and stored at −80°C until further analysis. At these time‐points, also forelimb grip strength was measured with a calibrated grip strength tester (Bioseb, Vitrolles) as described previously [25]. To calculate relative mean grip strength, absolute mean grip strength was divided by body weight.

#### 2.2.5 Postmortem Tissue Processing

At experimental endpoint, animals were sacrificed by cervical dislocation. Cecum content was collected into screw cap vials, snap‐frozen in liquid nitrogen and stored at −80°C until further analysis. Skeletal muscles (i.e., m. soleus, m. plantaris, m. gastrocnemius, m. tibialis anterior (TA) and m. extensor digitorum longus (EDL)) were collected from both hind limbs, using standardized dissection methods, weighed in pairs on an analytical balance (Fisher Scientific) and snap‐frozen [26]. Spleens were isolated, weighed, and transferred into 5 mL of ice‐cold DMEM. Single‐cell suspensions were prepared from spleens utilizing a gentleMACS dissociator (Miltenyi Biotec B.V., Leiden) and passing through a 70 µm pore cell strainer (Greiner Bio‐one, Alphen aan den Rijn). After centrifugation, cell pellets were resuspended in freezing medium (85% FBS, 10% DMSO (Sigma‐Aldrich, Zwijndrecht) and 5% of 50% glucose (Braun Melsungen AG, Melsungen) and transferred to liquid nitrogen for prolonged storage until required for analysis.

### 2.3 rRNA Sequencing

#### 2.3.1 DNA Isolation

Fecal DNA isolation was performed at BioVisible‐Microbial Diagnostics (Groningen) using the Fast DNA Stool Mini Kit (Qiagen 51604, Venlo). Samples were bead (0.1 mm glass) beaten three times 30 s (using the FastPrep‐24 instrument (program 5.5), with 5‐min incubations on ice in between. DNA was eluted in a final volume of 200 µL, and DNA concentrations were measured using a Qubit 4 fluorometer (Thermo Fisher Scientific, Walham).

#### 2.3.2 Gene Amplicon Sequencing

The V3‐V4 regions of the bacterial 16S rRNA gene were PCR amplified using a single reaction workflow. Following manufacturer's instructions simultaneous indexing and target amplification was performed with EasySeq 16S Microbiome Library Prep Kit (NimaGen, Nijmegen). Resulting amplicon libraries were sequenced on an Illumina MiSeq instrument (Illumina, Eindhoven) using the MiSeq Reagent Kit v3 (2 × 300 cycles, 10% PhiX) to produce paired‐end reads of 300 bases.

### 2.4 SCFA Analysis

SCFA levels in homogenized cecum content and fecal samples were quantitatively determined by a Shimadzu‐GC2010 gas chromatograph with a flame ionization detector (hydrogen as mobile phase, a ZB‐FFAB 15 m × 0.53 × 1.0 µm column and temperature program 90 till 220°C). A total of 25 µL sample was mixed with 175 µL internal standard mix containing 1% formic acid and 2‐ethylbutyric acid. After every 10 samples, the column was washed with 1 µL 1% (v/v) formic acid, followed by injection of 1 µL standard SCFA mix (2.5 mM acetic acid (Biosolve, Valkenswaard), 1.62 mM propionic acid (Merck, Zwijndrecht), 1.02 mM iso‐butyric acid (Merck, Zwijndrecht), 1.02 mM n‐butyric acid, 0.880 mM iso‐valeric acid, and 0.88 mM n‐valeric acid (Sigma‐Aldrich, Zwijndrecht) to monitor occurrence of memory effects. Sample SCFA concentrations were calculated based on a peak area constructed calibration curve.

### 2.5 Flow Cytometry Analysis of Splenocytes

Frozen splenocytes were thawed in a 37°C water bath and re‐suspended in pre‐warmed DMEM (10% FBS). After centrifugation and subsequent resuspension in cold PBS, cells were counted (CellDrop‐FL (DeNovix, Wilmington)). 2 × 10^6^ Cells were stained in a V‐bottom 96‐well transparent plate (Greiner Bio‐one, Alphen aan den Rijn), carefully maintaining them on melting ice throughout the staining procedure. First, cells were incubated with LIVE/DEAD Fixable Aqua Dead Cell Stain kit (L/D Aqua, Life Technologies, Carlsbad). Subsequently, cells were rinsed with fluorescence‐activated cell sorting (FACS) buffer, incubated with anti‐CD16/32 antibodies (2.4G2, BD Biosciences, San Diego) to block Fc receptors, and stained with a panel of specific surface marker antibodies for subsequent flow cytometry analysis (Table S2). Last, cells were fixated with 1% PFA and measured the next day. For each fluorophore, single stained cells were utilized to determine fluorescence compensation, and gating strategy was optimized by incorporating Fluorescence Minus One samples. Data were acquired on the FACS Canto II (BD Biosciences, San Diego) utilizing an 8‐color panel, with the acquisition process being controlled by FacsDIVA V.6.1.2 software (BD Biosciences, San Diego). Gating strategy is shown in Figure S1. Analysis of acquired data was carried out using FlowJo V.10.0.8 software (Tree Star, Ashland).

### 2.6 Data and Statistical Analysis

#### 2.6.1 16S rRNA Sequencing Data Pre‐Processing and Analysis

Data preprocessing was performed in R v4.3.1. Forward (FW) and reverse (RV) primer sequences were removed using cutadapt v4.4 [27]. Paired‐end reads were trimmed (FW = 240 bases and RV = 210 bases), denoised (default settings), and merged (minOverlap = 10, maxMismatch = 0). Chimeras were identified and removed, and an amplicon sequence variant (ASV) table was constructed. ASVs with lengths of < 350 or > 500 bases were discarded and remaining ASVs were annotated using the DADA2 implementation of the naïve Bayesian classifier based on the SILVA v138.1 reference database [28]. Following, data analysis was done using R v4.3.1 within R Studio v2023.06.2+561 (Boston, MA) and visualized using R package ggplot2 v3.4.2. Statistical analysis was 2‐sided and *p* values were corrected for multiple testing using Benjamini–Hochberg method unless otherwise stated. Samples with < 10 000 raw reads and mice with missing samples at one or more time‐points were excluded. In addition, ASVs with < 10% prevalence in all samples were filtered out. Shannon diversity was computed using R package vegan v2.6‐4. To ensure scale‐invariance and taking into account the compositional nature of sequencing data, we modeled the probability of the count data using the Dirichlet distribution (128 Monte Carlo iterations) performed with the ALDEx2 R package v.1.26.0. Probabilities were center log ratio (clr) transformed [29] and subsequently visualized using Principal Component Analysis with microViz v0.10.1 R‐package. Permutational analysis of variance (PERMANOVA) was performed on Aitchison distance matrix using the R package vegan v2.6‐6.1 Differential abundance analysis was performed using R package LinDA v 0.1.0 [30] with Bonferroni correction and the rest under default settings. Heatmaps with log2fold changes and hierarchical clustered rows were generated using ComplexHeatmap R package v2.10.0.

#### 2.6.2 Other Statistical Tests

No animals were excluded from any analysis. All parameters are reported as mean ± SD. To analyze therapeutic impacts of different diets, multivariate analysis of PFS was performed using Cox Proportional Hazards models with treatment group as a categorical variable (StataSE 17). Time to local failure was defined as reaching 4xSV and cure was defined as no palpable tumor for 60 consecutive days. Results are reported as hazard ratios (HRs) and significant HRs < 1 indicate that experimental diet(s) provides greater therapeutic effects as compared to composition 1. Proportional hazards assumption was tested based on the distribution of Schoenfeld residuals and was not violated for any of the models. Differences in average food intake, muscle weight, tumor take, spleen weight, cecal SCFA levels, and flow cytometry data were tested for normality using the Shapiro–Wilk test. For normally distributed data, statistical differences between diets were analyzed using one‐way ANOVA with Tukey's multiple comparisons test post‐hoc correction, and differences between cured and non‐cured mice were tested with a parametric *t*‐test. For data that did not pass normality testing, statistical differences between diets were analyzed using a non‐parametric Kruskal–Wallis multiple comparison test with Dunn's multiple comparison test post‐hoc correction and differences between cured and non‐cured mice were tested using a Mann–Whitney test. If significant differences between cured and non‐cured mice were found, a logistic regression model was used to correct for diet. Longitudinal grip strength tests and SCFA levels in fecal samples collected over time were compared between diets using mixed‐effects analysis with Tukey's post‐hoc correction, and between time‐points using a mixed‐effects analysis with Dunnets post‐hoc correction. To compare cured and non‐cured mice in the longitudinal samples, a mixed‐effects analysis with Sidak's post‐hoc correction was applied. To test for correlations between SCFAs and immune cells, Spearman's rank correlation coefficients or Pearson correlation coefficients analysis were performed depending on results of the normality testing. Statistical analyses were performed using GraphPad Prism Software v8.0.2 and IBM SPSS Statistics 26. *p* values smaller than 0.05 were considered statistically significant and indicated as *p ≤ 0.05, **p ≤ 0.01, and ***p ≤ 0.001.

## 3 Results

### 3.1 Dietary Fiber Composition Modulates Therapeutic Outcome

**FIGURE 2:**
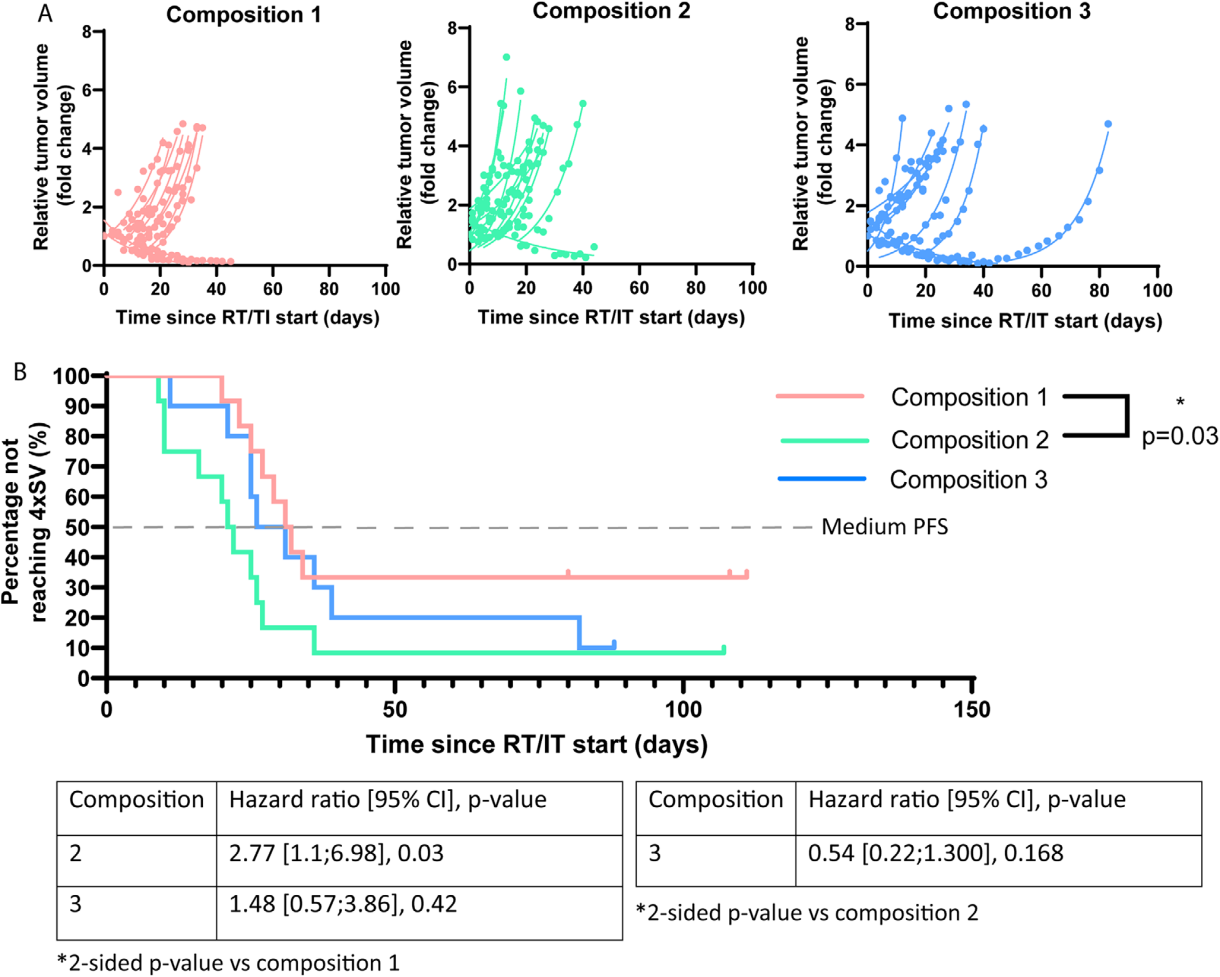
Fiber composition modulates therapeutic outcome. (A) Individual tumor growth curves normalized to starting volume (SV) followed until at least 4xSV was reached. Exponential tumor curves are shown from the lowest point after RT/IT treatment until tumor volume reaches at least 4xSV. For cured mice, curves are shown from SV until no tumor was palpable anymore. (B) Progression‐free survival curves and Cox regression analysis for different diets combined with RT/anti‐PD‐L1.

Tumor take (prior RT/IT) was not affected by fiber compositions (Figure S2). In 67.6% of the animals (23 out of 34) an initial tumor shrinkage was observed after RT/IT treatment, indicating concurrent RT/IT treatment exerts effects on tumor growth. Four animals on composition 1 did not experience tumor regrowth, compared to one animal in both other compositions. Diet composition 1 resulted in slower tumor growth compared to diet composition 2, with a medium PFS of 31.5 days compared to 21.5 days (HR 2.77 [1.1; 6.98], *p* = 0.03) (Figure 2A,B). Diet composition 3 did not modulate therapeutic outcomes compared to both compositions (*p* = 0.42 and *p* = 0.168) (Figure 2A,B).

### 3.2 Fiber Composition Does Not Affect Nutritional Status

To assess the impact of the diets on nutritional status, food intake, body weight, and muscle mass and strength were monitored. No loss of body weight, muscle mass, or muscle strength was observed (Figure S3). Average tumor mass in non‐cured animals was 5.1 ± 1.3% of body weight at endpoint. Fiber composition did not modulate average food intake values and food intake over time (data not shown). Fiber compositions did not modulate weights of the soleus, tibialis anterior, and extensor digitorum longus muscles. The plantaris and gastrocnemius muscles were slightly smaller for compositions 2 and 3, respectively (Figure S3C). Muscle masses were not different between cured/non‐cured animals (data not shown). Fiber composition did not modulate front limb muscle strength (Figure S3D).

### 3.3 Fiber Composition Modulates the Microbial Diversity and Composition

**FIGURE 3:**
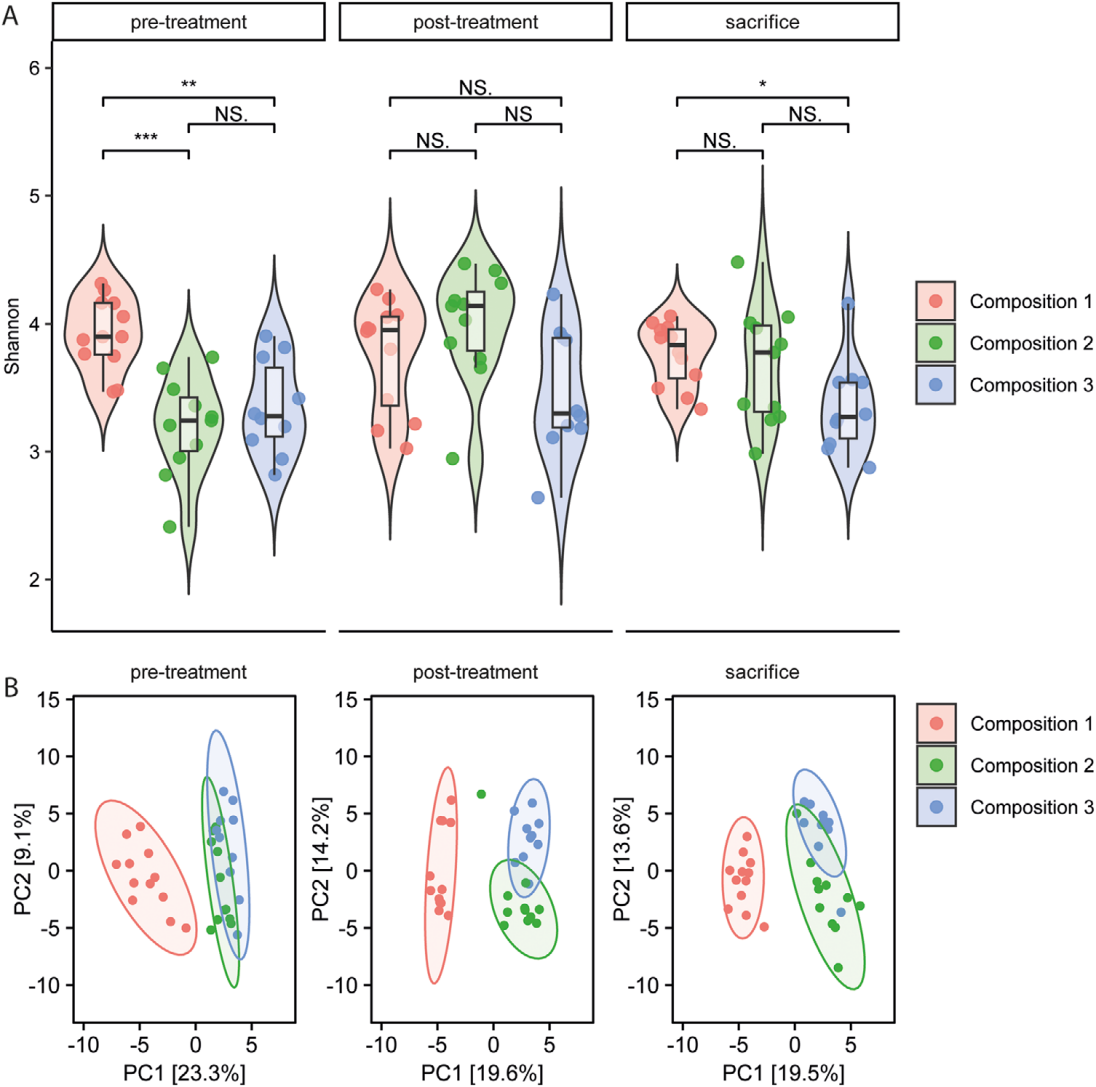
Fiber composition modulates gut microbiome composition. Shannon index scores (A) and community structure (principle component analysis) (B) between dietary compositions at different time points.

To investigate the impact of our fiber compositions on the gut microbiota, sequencing of the 16S rRNA V3‐V4 hypervariable gene regions was performed on fecal samples, collected at three time‐points (Figure 1). Prior RT/IT, compositions 2 and 3 had a lower microbial diversity (Shannon, *p* < 0.01) in fecal samples compared to composition 1. Microbial diversity (Shannon index) was not significantly different after RT/IT between the three compositions, while at sacrifice, diet composition 3 resulted again in a lower diversity compared to composition 1 (*p* < 0.01) (Figure 3A). PERMANOVA indicated that the microbiota community structures (between‐sample Aitchison distance) in the fecal samples from the animals receiving the respective diets were statistically significantly different at all three time‐points (*p* = 0.002 and *p* = 0.004) (Table S3). Samples from animals receiving composition 2 or 3 clustered together in the principle component analysis (PCA) and this clustering was observed at all three time‐points (Figure 3B), however, after correction for multiple testing, PCA indicated that samples from animals receiving compositions 2 and 3 had different microbiota community structures (between‐sample Aitchison distance) at all three time‐points (Table S3).

**FIGURE 4:**
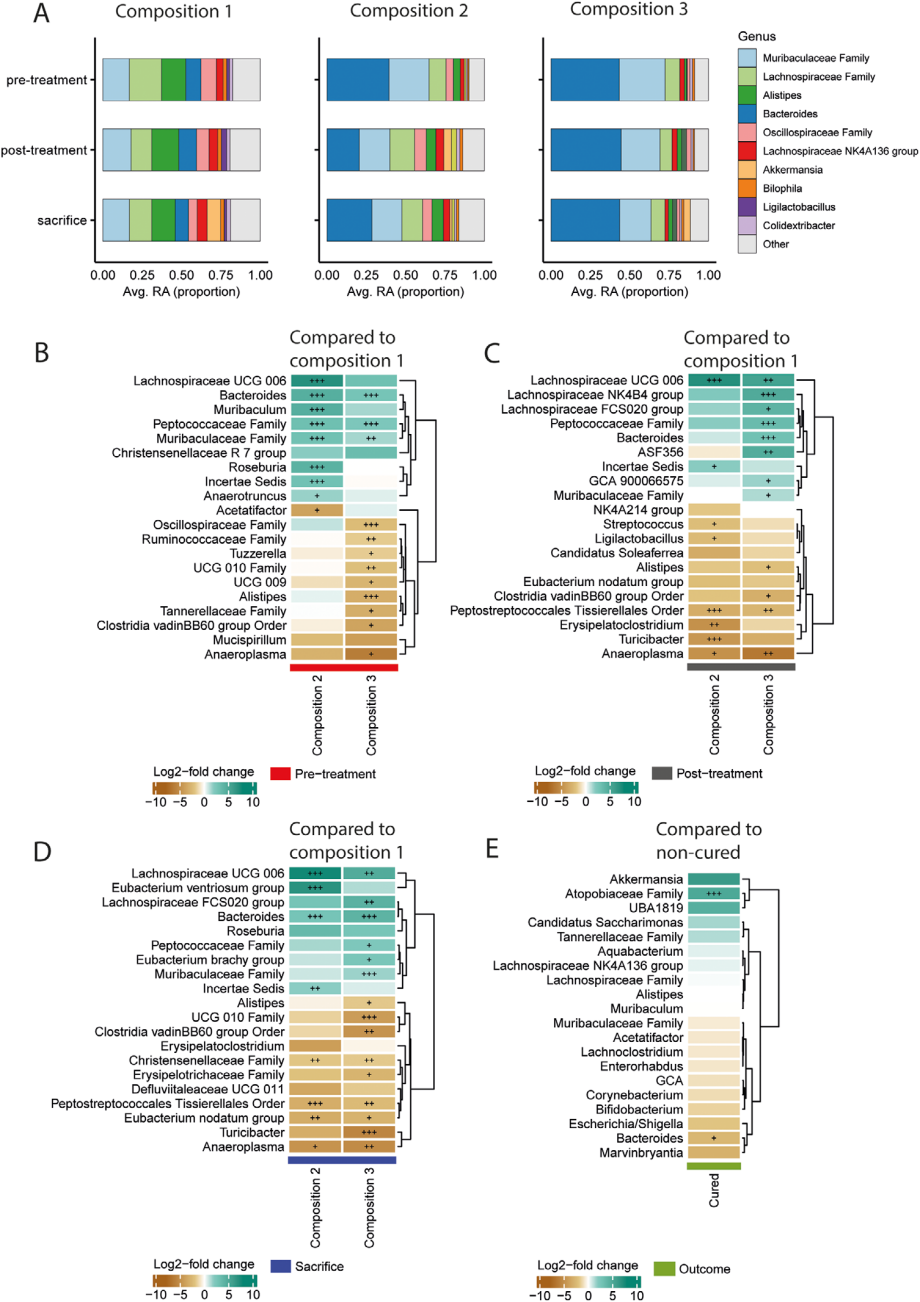
Detailed analysis of gut microbiome composition changes. (A) Average relative abundance (proportion) of 10 most prevalent genera in fecal samples. Heatmaps depicting the changes in genera comparing compositions 2 and 3 to composition 1, prior RT/IT (B), after RT/IT (C), and at sacrifice (D). Heatmap depicting changes in genera comparing samples from cured and non‐cured animals at sacrifice receiving composition 1 (E).

Following, we aimed to perform a detailed analysis of specific genera differences in fecal samples from animals receiving our different fiber compositions. First, the relative abundance of the 10 most abundant genera in the fecal samples was compared. In fecal samples from animals receiving compositions 2 and 3, there was a notable increase in the relative abundance of *Bacteroides*, across all three time‐points (Figure 4A).

To assess if these increases were significantly different, a differential abundance analysis with LinDA (Linear models for Differential Abundance analysis) was performed [30], comparing samples from animals receiving the respective compositions for each time‐point separately (pretreatment, posttreatment, and sacrifice). Before RT/IT, *Bacteroides, Peptococcacea Family*, and *Muribaculcaceae Family* were significantly enriched in both compositions 2 and 3, compared to composition 1. Further, in composition 2, *lachnospiraceae UCG 006, Muribaculum, Roseburia*, and *Anaerotruncus* were significantly enriched compared to composition 1, while *Acetatifactor* was depleted. Comparing compositions 1 and 3, in composition 3 *Oscillospiraceae Family, Ruminococcaceae Family, Tuzzerella, UCG 10 Family, UCG 009, Alistipes, Tannerellaceae Family, Clostriduea vadinBB60 group* and *Anaeroplasma* were depleted (Figure 4B). Comparing compositions 2 and 3 prior RT/IT treatment, in composition 3 *Acetatifactor and Erysipelatoclostridium* were enriched, while *Alistipes, Oscillospiraceae Family*, and *Roseburia* were depleted (Figure S4A). Post RT/IT treatment different heatmap patterns were observed, with enrichment of *Lachnospiraceae UCG 006* and depletion of *Anaeroplasma* in both compositions 2 and 3, compared to composition 1. Additionally, compared to composition 1, in composition 2*, Streptococcus, Ligilactobacillus, Peptostreptococcales Tissierellales Order, Erysipelatoclostridium, Turicibacter* and *Anaeroplasma* were depleted. In composition 3, *Lachnospiraceae NK4B4 group* and *FCS020 group*, *Peptococcaceae Family, Bacteroides, ASF356, GCA 900066575* and *Muribaculaceae Family* were enriched, while *Alistipes, Eubacterium nodatum group, Clostridia vadinBB60 group Order* and *Anaeroplasma* were depleted (Figure 4C). Comparing compositions 2 and 3 with each other post‐treatment, only *ASF356* was enriched in composition 3 (Figure S4B). At sacrifice, again it was observed that compared to composition 1, *Lachnospiraceae UCG 006* was enriched, and *Anaeroplasma* depleted in both compositions 2 and 3. In addition, *Bacteroides* was enriched, and *Christensenellaceae Family, Peptostreptococcales Tissierellales Order* and *Eubacterium nodatum group* were depleted in compositions 2 and 3, compared to composition 1. Furthermore, only in composition 2, it was observed that *Eubacterium ventriosum group* was enriched, and *Christensenellaceae Family* were depleted compared to composition 1. In composition 3, only *Lachnospiraceae FCS020 group, Peptococcaceae Family Eubacterium brachy group* and *Muribaculaceae Family* were enriched, while *Alistipes, UCG 010 Family, Clostridia vadinBB60 group Order, Erysipelotrichaceae Family* and *Turicibacter* were depleted compared to composition 1 (Figure 4D). Comparing compositions 2 and 3 at sacrifice, it was observed that several genera were depleted in composition 3, including *UCG 010 Family, Clostridia vadinBB60 group Order, Alistipes, Oscillospiraceae Family*, and *Eubacterium ventriosum group* (Figure S4C).

### 3.4 Enrichment of *Atopobiaceae Family* and Depletion of *Bacteroides* in Fecal Samples From Cured Animals

To investigate whether specific genera could be associated with therapeutic outcomes distinguishing between cured and non‐cured mice, subgroup analysis within composition 1 with fecal samples collected at sacrifice was performed. It was observed that *Atopobiaceae Family* was absent in non‐cured animal and significantly enriched in the samples from the cured mice (Figures 4E and S5A). Furthermore, *Bacteroides* was depleted in the samples from the cured animals compared to the non‐cured animals in this subgroup analysis (Figures 4E and S5B). Similar subgroup analysis within compositions 2 and 3 were not possible, since in both groups only one animal developed curative responses.

### 3.5 Comparable SCFAs Levels in Cecum and Feces Between Cured and Non‐Cured Mice

**FIGURE 5:**
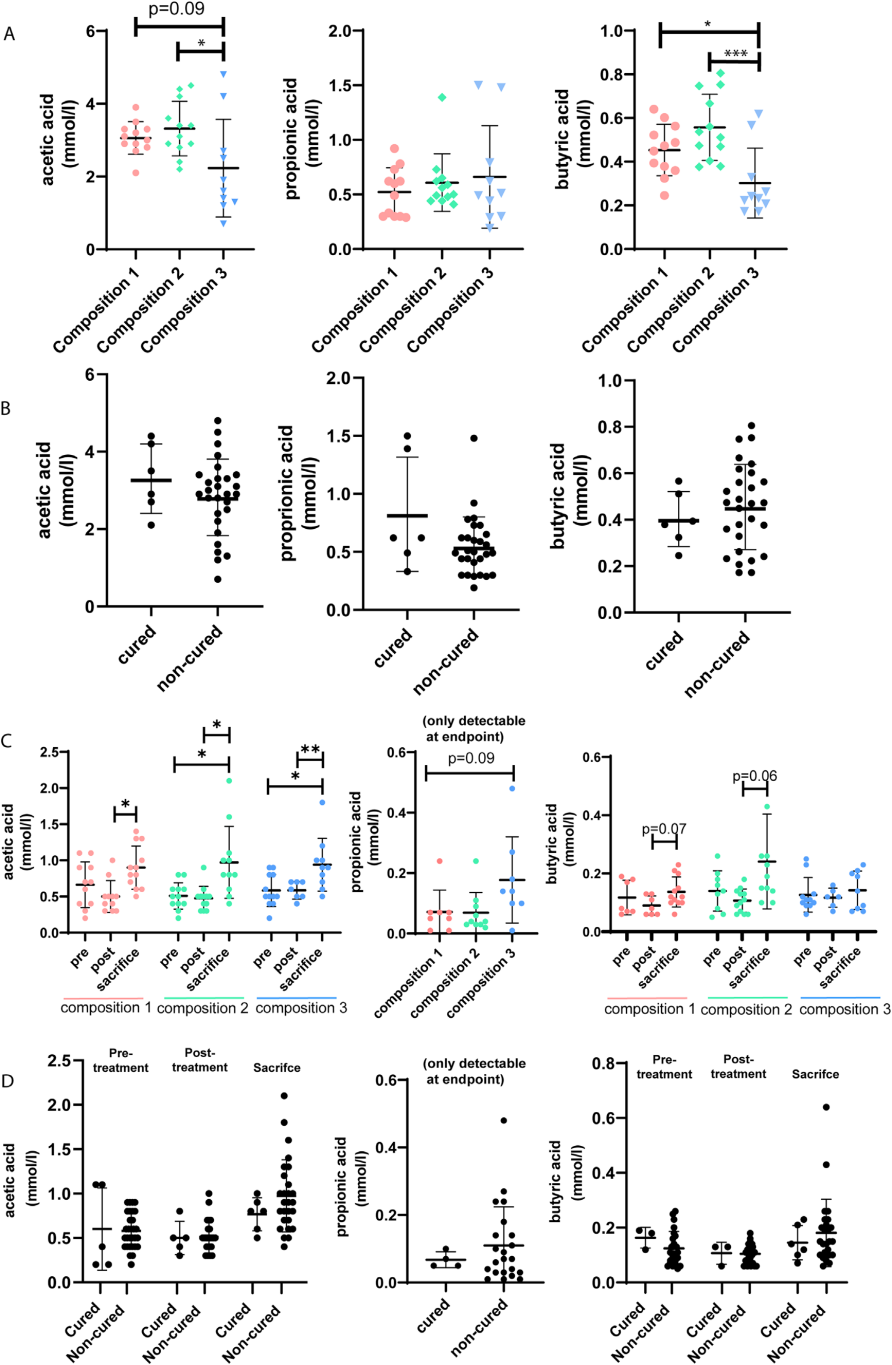
SCFAs levels in cecum and fecal samples. (A) Acetic, propionic and butyric acid levels in cecum content for different compositions and (B) cured/non‐cured mice. (C) Acetic and butyric acid levels in fecal samples collected over time, and propionic acid levels in fecal samples at sacrifice for different compositions (C) and cured/non‐cured mice (D).

SCFA levels in cecal and fecal samples were quantified to explore whether levels of these metabolites were related to differential therapeutic outcomes between different fiber compositions. Acetic acid levels in cecal samples from animals receiving composition 3 were lower compared to composition 2 (*p* = 0.02). Propionic acid levels in the cecum content did not differ between the fiber compositions (Figure 5A). Butyric acid was lower (*p* < 0.05) in the cecum content of animals receiving composition 3, compared to those receiving compositions 1 and 2 (Figure 5A). SCFA levels in the cecum content were similar in cured and non‐cured animals (Figure 5B). In fecal samples, acetic‐, butyric‐, and propionic acid levels were comparable between the fiber compositions. Toward sacrifice, increased acetic acid levels were observed, independent of the fiber composition, and for butyrate a positive trend was visible toward sacrifice in compositions 1 and 2, but not in composition 3. Propionic acid was only detectable in samples collected at sacrifice (Figure 5C). Between cured and non‐cured animals, there were no differences in SCFA levels in fecal samples prior‐ or after RT/IT, nor at sacrifice (Figure 5D).

### 3.6 Cured Mice Have Smaller Spleens With Less MDSCs

**FIGURE 6:**
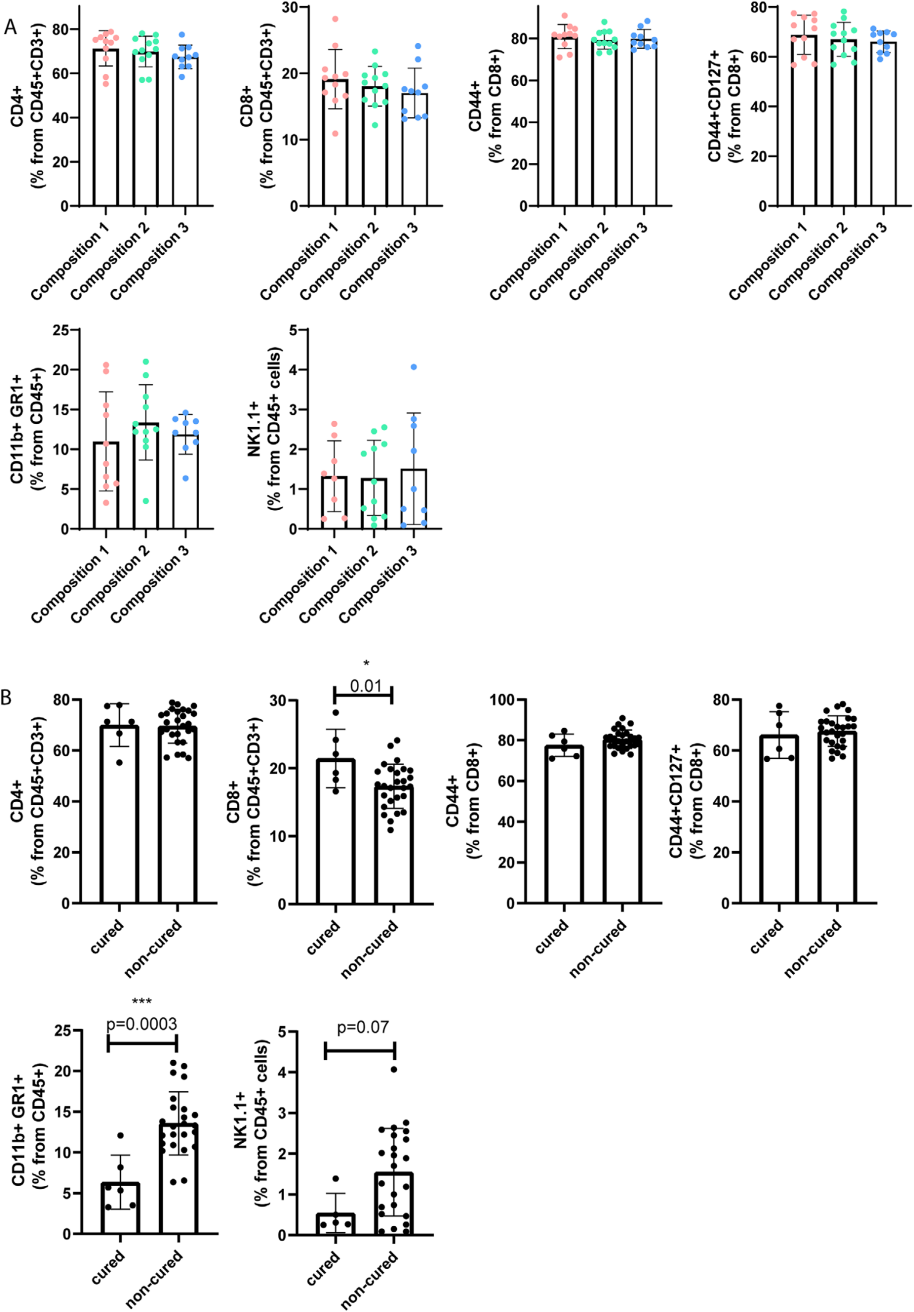
Immunological profiling in splenic cells. (A) Proportions of splenic T‐cells, natural killer (NK) cells and myeloid‐derived suppressor cells (MDSC) for different fiber compositions and (B) cured/non‐cured mice.

We next investigated whether the fiber compositions resulted in changes in splenic immune cells. Diets did not affect wet spleen weights (Figure S6), nor proportions of splenic T‐cell sub‐populations, natural killer (NK) cells or myeloid‐derived suppressor cells (MDSC) (Figure 6A). As specific SCFAs can interact with certain immune cells causing either pro‐ or anti‐inflammatory effects, correlations between SCFAs and immune cells were calculated. Acetic‐ and butyric acid levels in fecal samples collected at sacrifice correlated negatively with the proportion of CD4+ T‐cells (*p* = 0.025 and 0.028, respectively) and acetic acid levels correlated positively with the MDSC (*p* = 0.002) in these samples. In the cecal samples, acetic‐ and propionic acid levels correlated positively with CD8+ cytotoxic T‐cells (*p* = 0.042 and *p* = 0.0041, respectively), and propionic acid in the cecal samples correlated negatively with NK cells (*p* = 0.007) (Table 1). Subgroup analysis, comparing spleen weight and immune profile between cured and non‐cured mice indicated reduced spleen weights in cured mice (*p* < 0.001) (Figure S5), and their spleens contained relatively more CD8+ cytotoxic T‐cells (*p* < 0.05) and fewer MDSCs (*p* < 0.001) compared to non‐cured mice (Figure 6B). After logistic regression to correct for diet, spleen weight (*p* = 0.045) and MDSCs (*p* = 0.018), but not the number of CD8+ cytotoxic T‐cells (*p* = 0.064), remained predictive of therapeutic outcome (cured).

**TABLE 1:** Correlations between SCFAs and splenocytes.

		CD4+	CD8+	CD44+	CD44+ CD127+	MDSC	NK
Acetic acid fecal sample pretreatment	Correlation coefficient	−0.063	−0.212	0.239	0.189	0.057	0.321
	Sig. (2‐tailed)	0.733	0.244	0.187	0.301	0.770	0.096
	*N*	32	32	32	32	29	28
Acetic acid fecal sample posttreatment	Correlation coefficient	−0.098	0.099	−0.171	−0.214	−0.086	0.162
	Sig. (2‐tailed)	0.611	0.610	0.375	0.265	0.676	0.439
	*N*	29	29	29	29	26	25
Acetic acid fecal sample endpoint	Correlation coefficient	**−0**.396^*^	−0.131	0.113	−0.112	**0**.556^**^	0.102
	Sig. (2‐tailed)	**0**.**025**	0.473	0.540	0.542	**0**.**002**	0.604
	*N*	**32**	32	32	32	**29**	28
Butyric acid fecal sample pretreatment	Correlation coefficient	0.193	0.076	0.235	0.158	−0.068	−0.090
	Sig. (2‐tailed)	0.365	0.723	0.269	0.461	0.762	0.699
	*N*	24	24	24	24	22	21
Butyric acid fecal sample posttreatment	Correlation coefficient	−0.244	0.100	−0.154	−0.267	−0.255	0.172
	Sig. (2‐tailed)	0.251	0.641	0.472	0.207	0.265	0.456
	*N*	24	24	24	24	21	21
Butyric acid fecal sample endpoint	Correlation coefficient	**−0**.395^*^	0.091	0.167	−0.029	0.152	−0.061
	Sig. (2‐tailed)	**0**.**028**	0.628	0.370	0.875	0.439	0.763
	*N*	**31**	31	31	31	28	27
Propionic acid fecal sample endpoint	Correlation coefficient	−0.046	−0.158	−0.032	−0.156	0.018	0.286
	Sig. (2‐tailed)	0.825	0.452	0.879	0.455	0.936	0.186
	*N*	25	25	25	25	23	23
Acetic acid cecal sample	Correlation coefficient	0.309	**0**.361^*^	−0.176	0.133	0.065	−0.127
	Sig. (2‐tailed)	0.085	**0**.**042**	0.334	0.468	0.737	0.518
	*N*	32	**32**	32	32	29	28
Butyric acid cecal sample	Correlation coefficient	0.123	0.107	0.007	0.039	0.353	0.074
	Sig. (2‐tailed)	0.501	0.560	0.971	0.834	0.060	0.708
	*N*	32	32	32	32	29	28
Propionic acid cecal sample	Correlation coefficient	0.179	**0**.363^*^	−0.247	−0.079	0.053	**−0**.498^**^
	Sig. (2‐tailed)	0.326	**0**.**041**	0.173	0.668	0.787	**0.007**
	*N*	32	**32**	32	32	29	**28**

*Note*: Spearman's rank correlation coefficients or Pearson correlation coefficients were calculated depending on the results of the normality testing.

Abbreviations: MDSC, myeloid‐derived suppressor cells; NK, natural killer cells. *p*‐values smaller than 0.05 were considered statistically significant and indicated as *p≤0.05, **p≤0.01, and ***p≤0.001.

## 4 Discussion

Despite associations between increased fiber intake and improved RT and IT outcomes, it remains unknown whether dietary fiber composition influences combined RT/IT outcomes. This study is the first to explore whether dietary fiber composition influences therapeutic outcome of combined RT/ICI treatment. Our findings propose that dietary fiber composition influences therapeutic outcome of combined RT/IT in a preclinical subcutaneous CT26 colon cancer mouse model.

Biological effects of dietary fibers are influenced by their source, purity, and physicochemical properties like solubility, fermentability, viscosity, water binding capacity, type of monosaccharides and glycosidic bonds, chain length and intra/interchain linkages. These properties, amongst others, determine prebiotic potential and amount of SCFAs produced upon fermentation, with effects on the immune system and other mechanisms relevant for RT/IT efficacy. Composition 1 is based on cellulose which mainly functions as a bulking agent due to its non‐digestible/non‐fermentable nature [31]. In contrast, compositions 2 and 3 contain fermentable fibers that are expected to exert different effects on RT/IT outcomes, through direct and indirect mechanisms. Composition 2 was formulated with fibers known to modulate the immune system and composition 3 incorporates four fibers commonly found in a healthy human diet. Based on distinct profiles of these three compositions, variation in their impact on RT/IT was anticipated. Our results demonstrated that composition 1 led to a longer PFS compared to composition 2, with a key differentiator between the compositions being the fermentability of the fibers, suggesting partly a role for the gut microbiome in mediating these effects.

Multiple studies have explored the gut microbiota composition as a potential biomarker for predicting ICI efficacy. Although there is no consensus yet on the role of the microbiome (microbiota and their metabolites), a more diverse microbiome is generally considered advantageous [13]. Associations between fiber‐rich diets and improved responses to ICI and RT [18, 23], might partly be related to the fibers’ modulatory effects on the gut microbiota composition. In our study, compositions 2 and 3 did not enhance microbiota diversity, possibly because the fibers in these compositions may be metabolized selectively by specific microbes. Previously, it has been reported that increased fiber intake can lead to a decreased microbial diversity [32, 33], because specific fiber‐degrading taxa tend to expand upon fiber‐rich diets. When dietary ingredients are utilized by a wide variety of microbes, the result is a modest growth of many different microbial species, however, when specific fibers are only metabolized by a few specific microbes, mainly these specific microbes experience growth, leading to a lower microbial diversity [32, 33]. This principle has previously also been demonstrated with fibers used in the tested compositions. For example, healthy mice fed an oat beta‐glucan‐enriched diet (present in composition 3) for 8 weeks exhibited reduced microbial diversity in colonic digesta compared to those on the AIN‐93 diet with corn‐starch [34]. Similarly, mice on a high‐fat diet (HFD) supplemented with arabinoxylan oligosaccharides (present in compositions 2 and 3) had lower fecal microbial diversity than those on HFD alone or control diet with corn‐starch [35]. Additionally, a study comparing cellulose and inulin intake in mice over 88 days found significantly higher microbial diversity in the cellulose group [36]. Beyond microbial diversity, also the gut microbiota community structure was explored. We observed different gut microbiota community structures between the three fiber compositions at all time‐points (prior RT, after RT/IT, and at sacrifice). Following, we explored whether specific microbes could be associated with therapeutic outcome. Current literature describes favorable profiles, characterized by the presence of various members from the *Lachnospiraceae* and *Ruminococcaceae* families, as well as species from the *Faecalibacterium*, *Akkermansia*, and *Bifidobacterium* genera, as being linked to improved ICI treatment outcomes [37, 38, 39, 40] and *Bacteriodes* has been linked to worse ICI outcome [13]. In line with these data, we observed that *Bacteriodes* was more abundant in fecal samples collected at sacrifice from non‐cured mice compared to cured mice. Moreover, we observed, for the first time, a positive association between abundance of *Atopobiaceae Family* (at sacrifice) and curative outcome of RT/IT treatment. Nonetheless, based on this cross‐sectional microbiome analysis at endpoint, we cannot validly say that there are clinical better results in composition 1. This is for the reason we lack power for responder versus non‐responder comparisons on a diet level, with only one cured animal in compositions 2 and 3, versus four in composition 1. In conclusion, identifying gut microbiota composition signatures associated with RT/IT efficacy remains challenging and further investigation into the specific mechanisms at play is essential to optimize dietary fiber compositions for therapeutic benefits.

SCFAs may also influence RT/IT efficacy. SCFAs function as histone deacetylase inhibitors [41], influencing the immune response [42, 43] and supporting gut barrier integrity [44]. Most literature points toward beneficial effects of SCFAs on ICI efficacy [22, 23, 45], although there is also a study reporting that butyrate reduced the efficacy of anti‐CTLA‐4 treatment [46]. These conflicting results may be attributed to variations in experimental design, differences in types of dietary fibers fermented into SCFAs, specific cancer models, and ICIs applied. Remarkably, in our cecal samples, but not the fecal samples, lower acetic, and butyric acid levels were observed in the animals receiving composition 3, while composition 3 contains fermentable fibers. A potential biological explanation for this observation might be that the fibers in composition 3 are slowly fermentable due to their high branch complexity, and as such are fermented in more distal parts of the colon, rather than in the cecum [47]. In fecal samples collected at sacrifice we observed higher SCFA levels, compared to samples collected prior or after RT/IT. Possibly, genera responsible for SCFA fermentation needed a running in period, but in our study, analysis between time‐point to evaluate whether this has appeared was not performed as all heatmaps are cross‐sectional. Between cured and non‐cured animals, no differences in SCFA levels in cecal and fecal samples were observed, suggesting that other mechanisms are involved in the differential therapeutic outcomes observed between the fiber compositions. To further dissect mechanisms at play and relevant metabolites, future research could incorporate shotgun metagenomics (RNAseq) and fecal metabolomics.

Fibers may influence RT/IT therapy efficacy via modulation of the immune response, either directly, for example, via binding to receptors on immune cells [48], or indirectly via specific microbiota and SCFAs, which can lead to pro‐ or anti‐inflammatory responses [42, 43]. While CD8+ cytotoxic T‐cells are crucial for combating tumor cells, MDSCs suppress immune responsiveness [49]. We observed positive correlations between cecal levels of acetic‐ and propionic acid and the proportion of CD8+ cytotoxic T‐cells in the spleen. Also, a positive correlation was observed between fecal acetic acid levels in samples collected at sacrifice and the proportion of MSDCs in the spleen. However, it remains unclear whether these correlations influenced therapeutic outcomes (cure); further experiments with controlled SCFA administration via the diet or drinking water would be necessary to investigate such effects, similarly to the methods of Zhang et al. [22]. Notably, cured mice in our study did not exhibit different cecal or fecal SCFA levels compared to non‐cured mice. However, cured mice did display an increased proportion of CD8+ T‐cells and a decreased proportion of MDSCs in their spleens, suggesting other factors, beyond SCFAs contribute to immune profiles in the spleen in our model. These findings raise the question whether observed changes in the spleen are causing curative effects or are consequential.

Nutritional status is also relevant to consider in context of RT/IT efficacy, as malnutrition, often induced by adverse effect of RT and IT, has been shown to negatively affect clinical outcomes [16]. Likewise, weight loss is also a sign of toxicity in mice [50], and therefore, body weight monitoring is essential to assess safety of dietary interventions. We observed no differences in body weight or food intake among the fiber compositions, indicating animals do not develop anorexia or cachexia within the experimental timeframe of the study. Thus, the tested fiber compositions did not affect body weight and food intake. Also, no differences were observed in muscle strength, however, we did observe that the plantaris and gastrocnemius muscle weights were lower for fiber compositions 2 and 3, respectively. As mice at this age still accrue muscle mass [51] and the animals of composition 1 remained longer in the experiment, this observation likely reflects differences in continuous age‐related increases in muscle growth, rather than direct effects of the diets. Together, our data suggests that our diets were well‐tolerated, and that effects of various fiber compositions on therapeutic outcome (cured) appear unrelated to changes in nutritional status in this model.

Although our study provides some intriguing insights into effects of different fiber compositions on RT/IT efficacy, there are also limitations. First, the sample size of this experiment is relatively small. This small sample size also limits an in‐depth responder versus non‐responder comparison (only 6 out of 34 animals were cured leading to limited power). Second, cage effects in microbiome studies are relevant because of coprophagy behavior of mice. An effective method to reduce cage effects and increase statistical power is to reduce the number of animals per cage [52]. In our study, four animals were housed together; however, cured animals were found in different cages, pointing toward limited cage‐effects on therapeutic outcome. Third, the subcutaneous CT26 model is suboptimal as it does not fully replicate the cellular and biochemical (e.g., extracellular matrix) characteristics of an orthotopically growing colon tumor. Yet, this model offered the advantage that our lab has extensive experience with it in combined RT/IT studies [7, 24] and the specific treatment regimen used here had already been established before [6]. While an orthotopic model would offer greater physiological relevance, its use would necessitate the use of more advanced imaging modalities for tumor irradiation and monitoring tumor growth, significantly increasing the complexity of the experimental setup. Fourth, it is relevant to consider the separate effects of our diets on tumor growth and the interactions between the diets and IT or RT alone, although these aspects were not investigated, as additional control groups would be required. Fifth, we cannot determine whether presence of the tumor, therapy, or a combination of both had feedback responses on the gut microbiome composition which may have modulated effects of the fiber compositions on therapeutic outcome (cured), as also addition control groups would be required. Sixth, a comparison of immune cell infiltration within the tumors across the different dietary groups is missing in this study. While such an analysis would have been informative, only immune profiling on the spleen was performed, as this allowed us to compare immune responses between cured and non‐cured animals. Since cured mice no longer presented with tumors, direct comparison of tumor‐infiltrating immune cells between these groups would not have been feasible. Finally, as the aim of this study was to establish and characterize whether dietary fiber composition influences combined RT/IT outcome in the subcutaneous CT26 model, the study is primarily descriptive in nature. Yet, our findings provide a solid foundation and rationale for future studies to dissect the underlying mechanisms in more detail.

Overall, beyond the previously reported relevance of the quantity of dietary fibers, our study suggests that the composition may also play a role in influencing therapeutic outcomes of combined RT/IT in a preclinical CT26 colon cancer mouse model. Therefore, future dietary intervention studies aiming at optimizing the therapeutic efficacy of combined RT/IT should, beyond the amounts of dietary fibers, also consider its composition.

## Funding

This work was funded by the NWO domain Applied and Engineered Sciences and Danone Research & Innovation, with additional financial support from Topsector Agri and Food. Grant No. 16485 NutrI2FIT: Strengthening Immune Fitness—a Nutritional solution to boost cancer ImmunoTherapy efficacy.

## Conflicts of Interest

M.v.D., J.B., and A.H. are employees of Danone Research & Innovation. L.J.D. reports, outside the submitted work, minority shares in LivingMed Biotech and Convert Pharmaceuticals. J.T. reports, outside the submitted work, minority shares in Convert Pharmaceuticals. The remaining authors declare not to have any conflicts of interest.

## Declaration of AI and AI‐Assisted Technologies in the Writing Process

During the preparation of this work, the authors used ChatGPT version 4 in order to improve the scientific English. After using this tool, the authors reviewed and edited the content as needed and took full responsibility for the content of the publication.

## Supporting information




**Supporting File 1**: mnfr70370‐sup‐0001‐FigureS1.tif.

**Supporting File 2**: mnfr70370‐sup‐0002‐FigureS2.tif.

**Supporting File 3**: mnfr70370‐sup‐0003‐FigureS3.tif.

**Supporting File 4**: mnfr70370‐sup‐0004‐FigureS4.tif.

**Supporting File 5**: mnfr70370‐sup‐0005‐FigureS5.tif.

**Supporting File 6**: mnfr70370‐sup‐0006‐FigureS6.tif.

**Supporting File 7**: mnfr70370‐sup‐0007‐SupMat‐Figure‐Legends.docx.

**Supporting File 8**: mnfr70370‐sup‐0008‐TableS1.docx.

**Supporting File 9**: mnfr70370‐sup‐0009‐TableS2.docx.

**Supporting File 10**: mnfr70370‐sup‐0010‐TableS3.docx.

## Data Availability

Sequencing data have been registered at the European Nucleotide Archive (ENA) under the following number: PRJEB80747.
