# Supplementary figures and images for: The Effect of Dietary Fiber Compositions on the Therapeutic Outcome of Combined Radio‐ and Immunotherapy in a Preclinical Cancer Model
Source: Mol Nutr Food Res. 2026 Jan 20;70(2):e70370. doi: 10.1002/mnfr.70370 (PMC12820406; doi:10.1002/mnfr.70370)

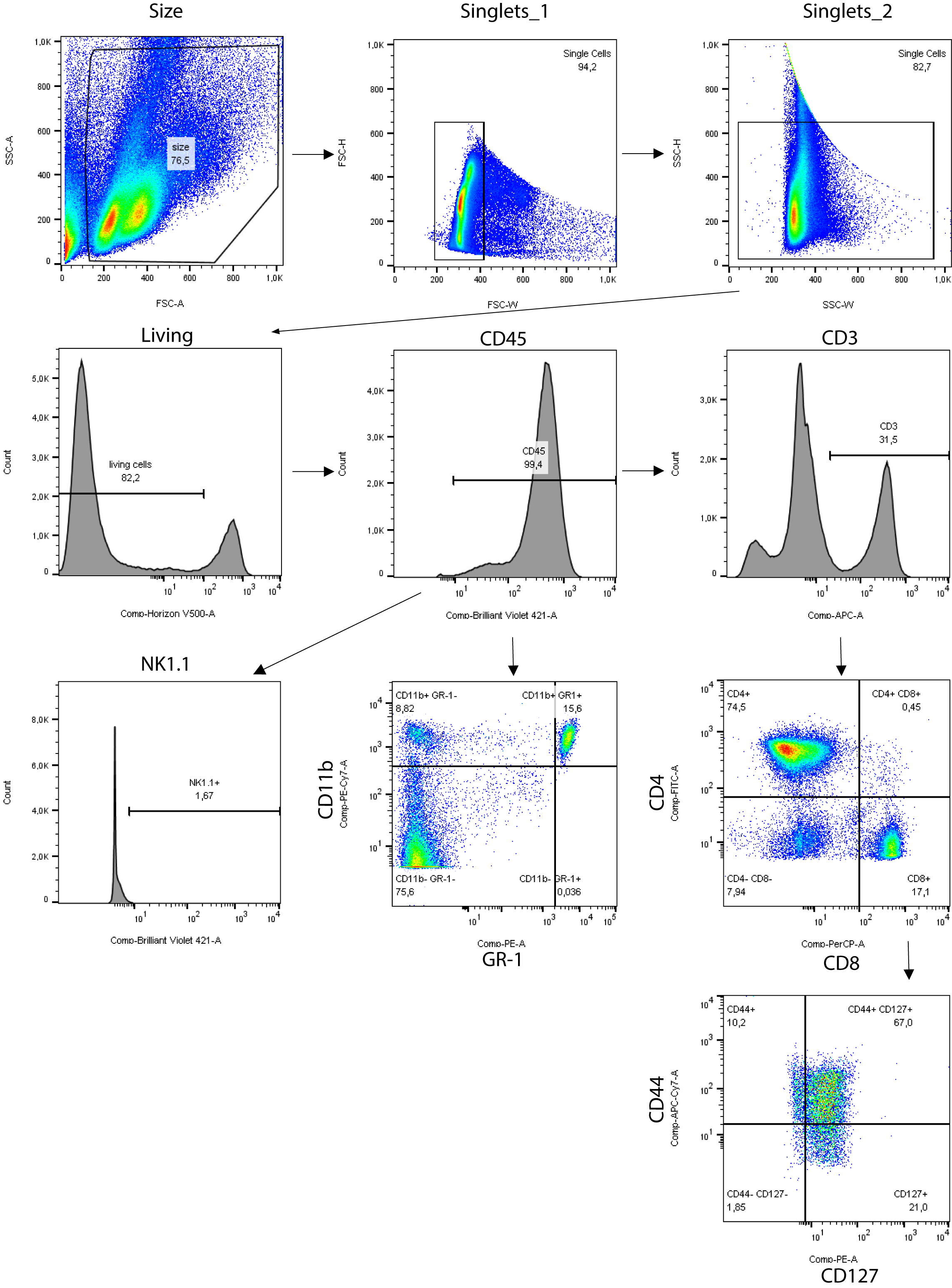

Supplement: Supplementary file 1 — Supporting File 1: mnfr70370‐sup‐0001‐FigureS1.tif. [file MNFR-70-e70370-s001.tif]

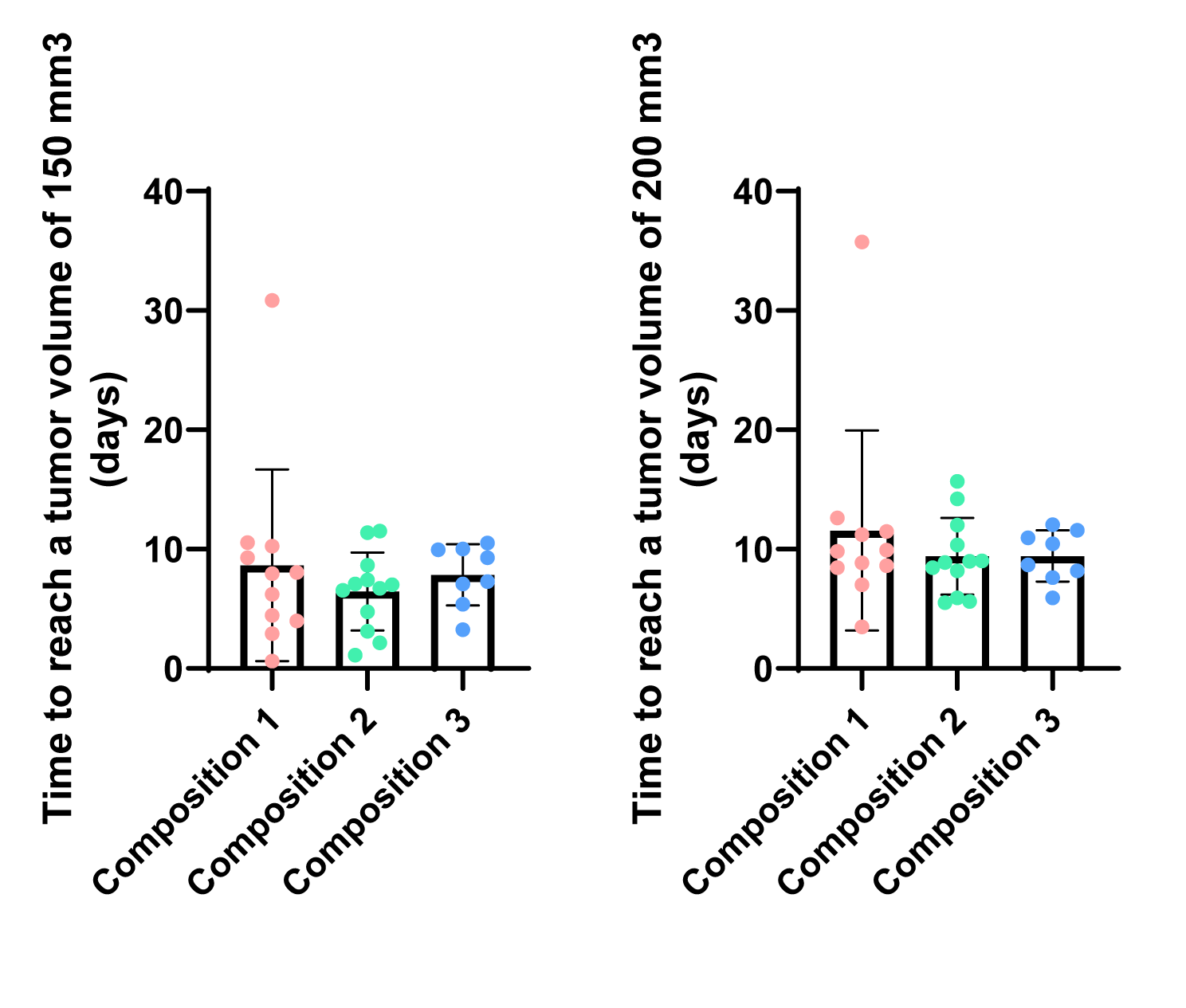

Supplement: Supplementary file 2 — Supporting File 2: mnfr70370‐sup‐0002‐FigureS2.tif. [file MNFR-70-e70370-s008.tif]

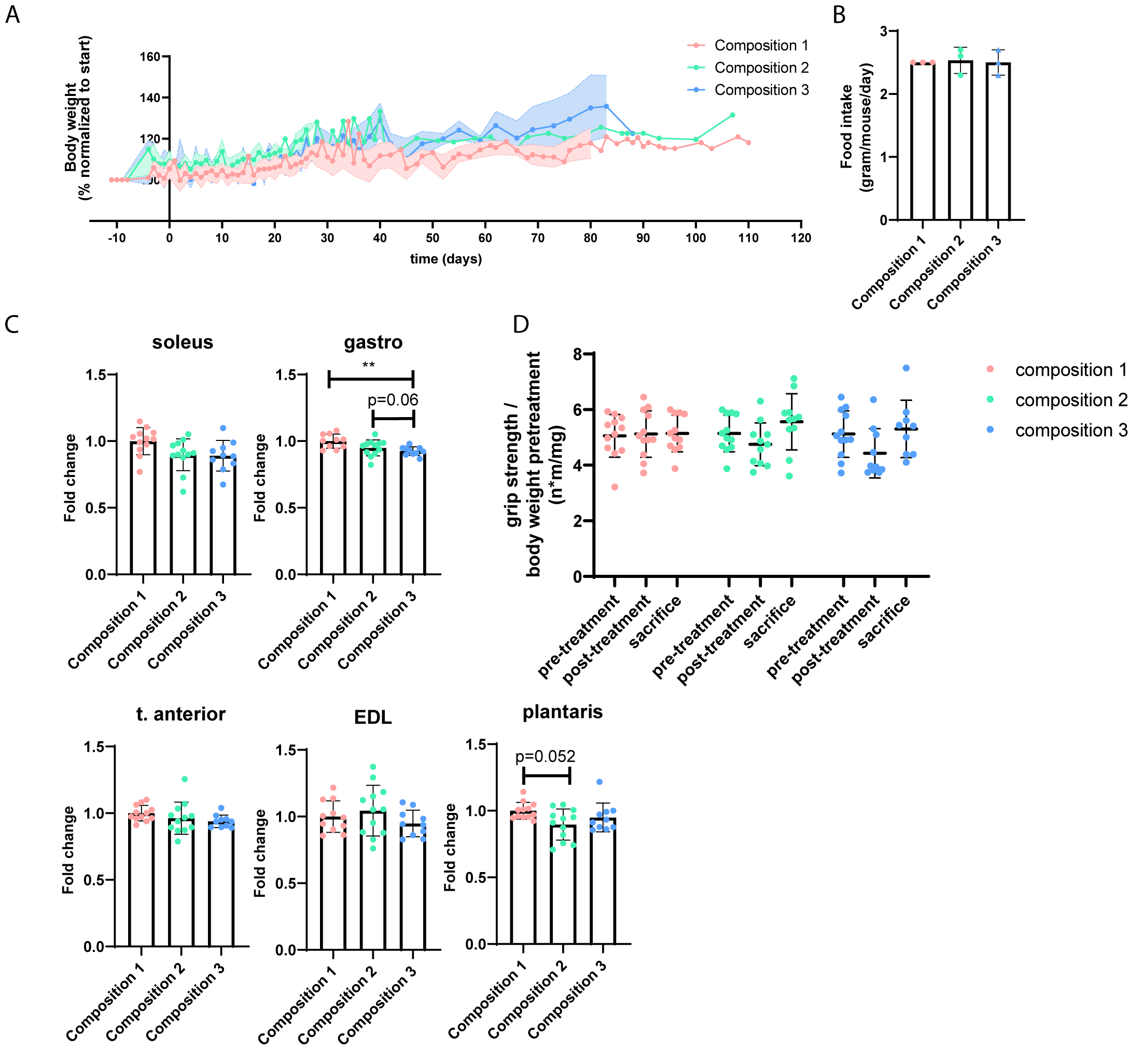

Supplement: Supplementary file 3 — Supporting File 3: mnfr70370‐sup‐0003‐FigureS3.tif. [file MNFR-70-e70370-s005.tif]

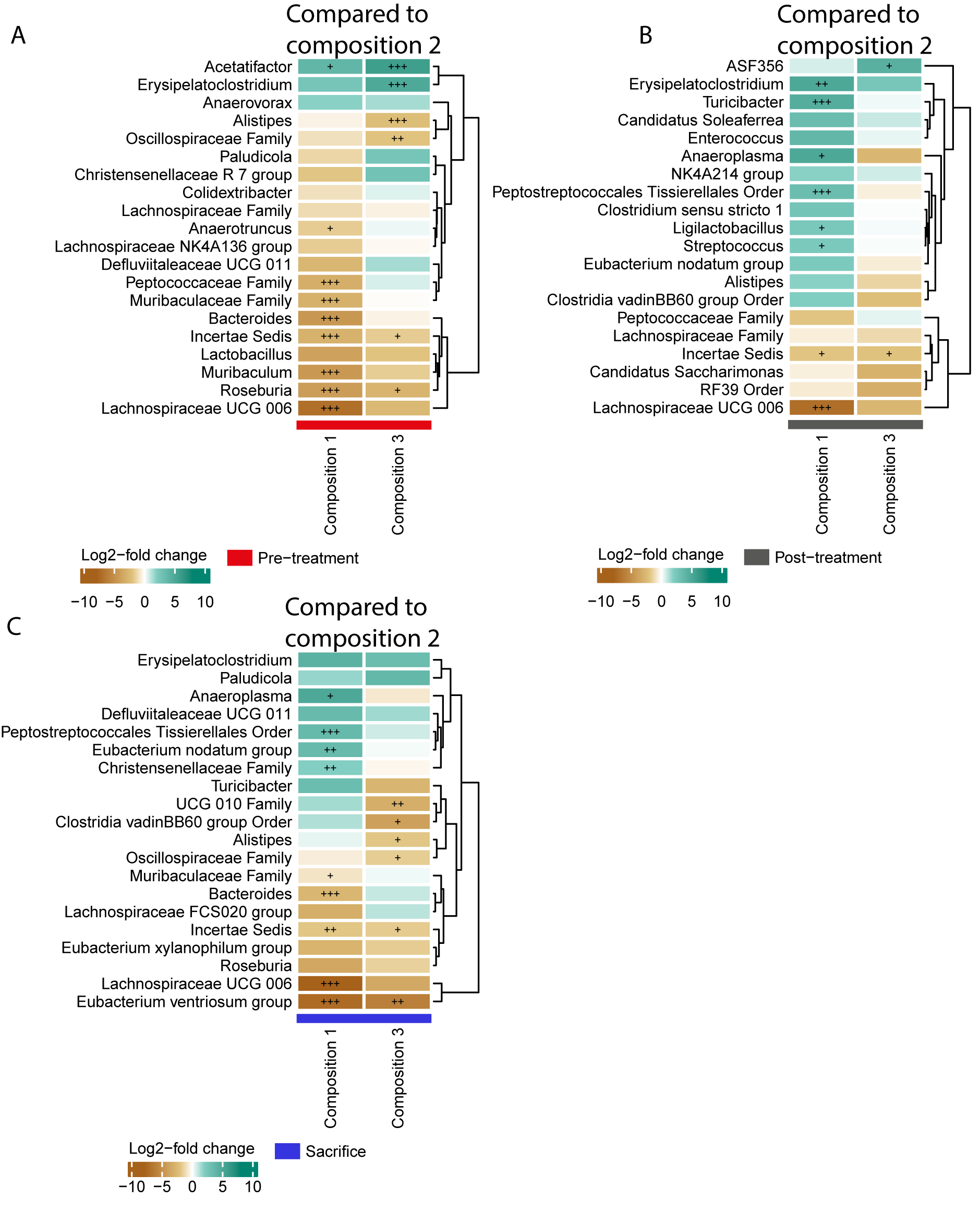

Supplement: Supplementary file 4 — Supporting File 4: mnfr70370‐sup‐0004‐FigureS4.tif. [file MNFR-70-e70370-s006.tif]

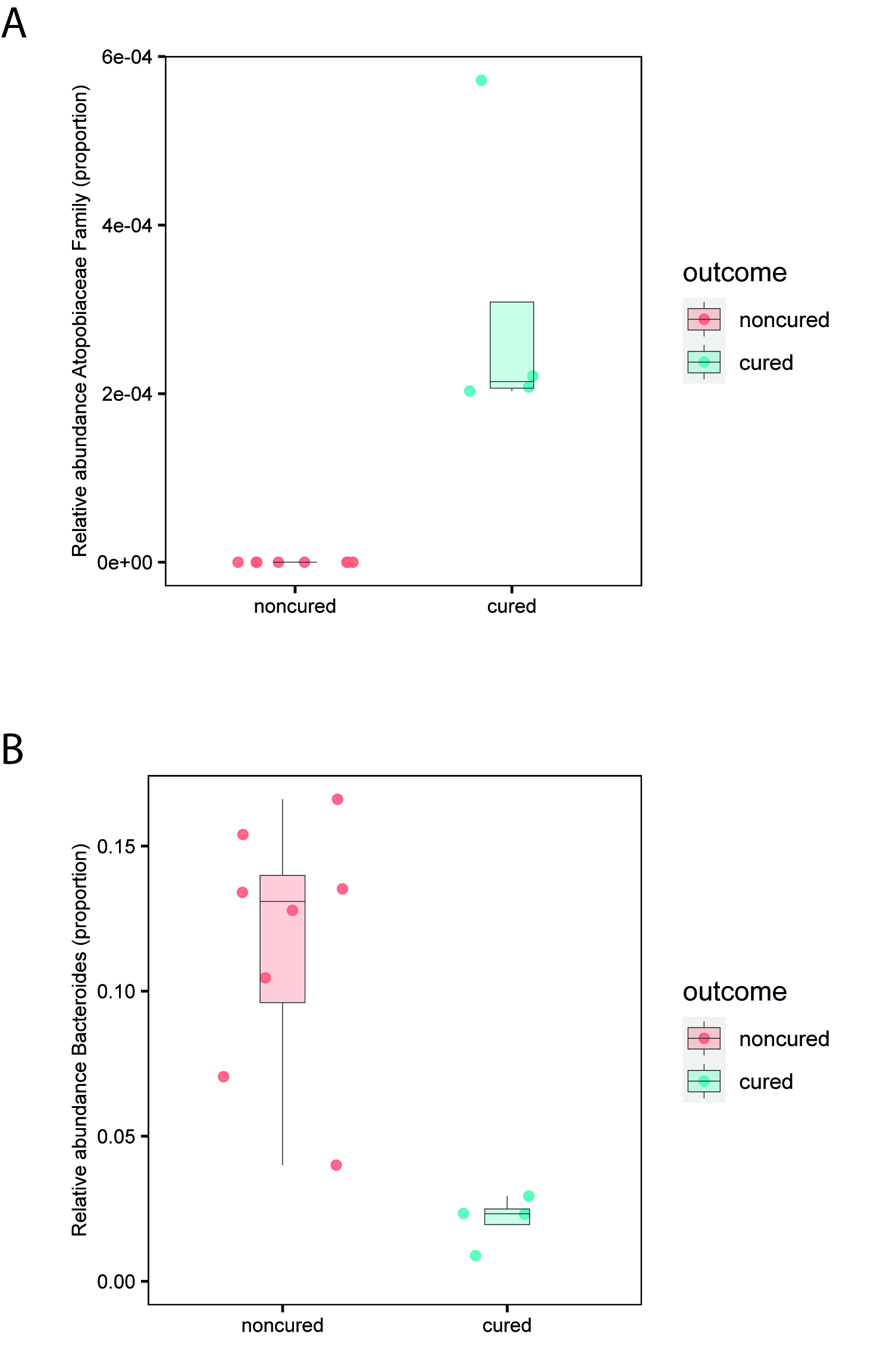

Supplement: Supplementary file 5 — Supporting File 5: mnfr70370‐sup‐0005‐FigureS5.tif. [file MNFR-70-e70370-s010.tif]

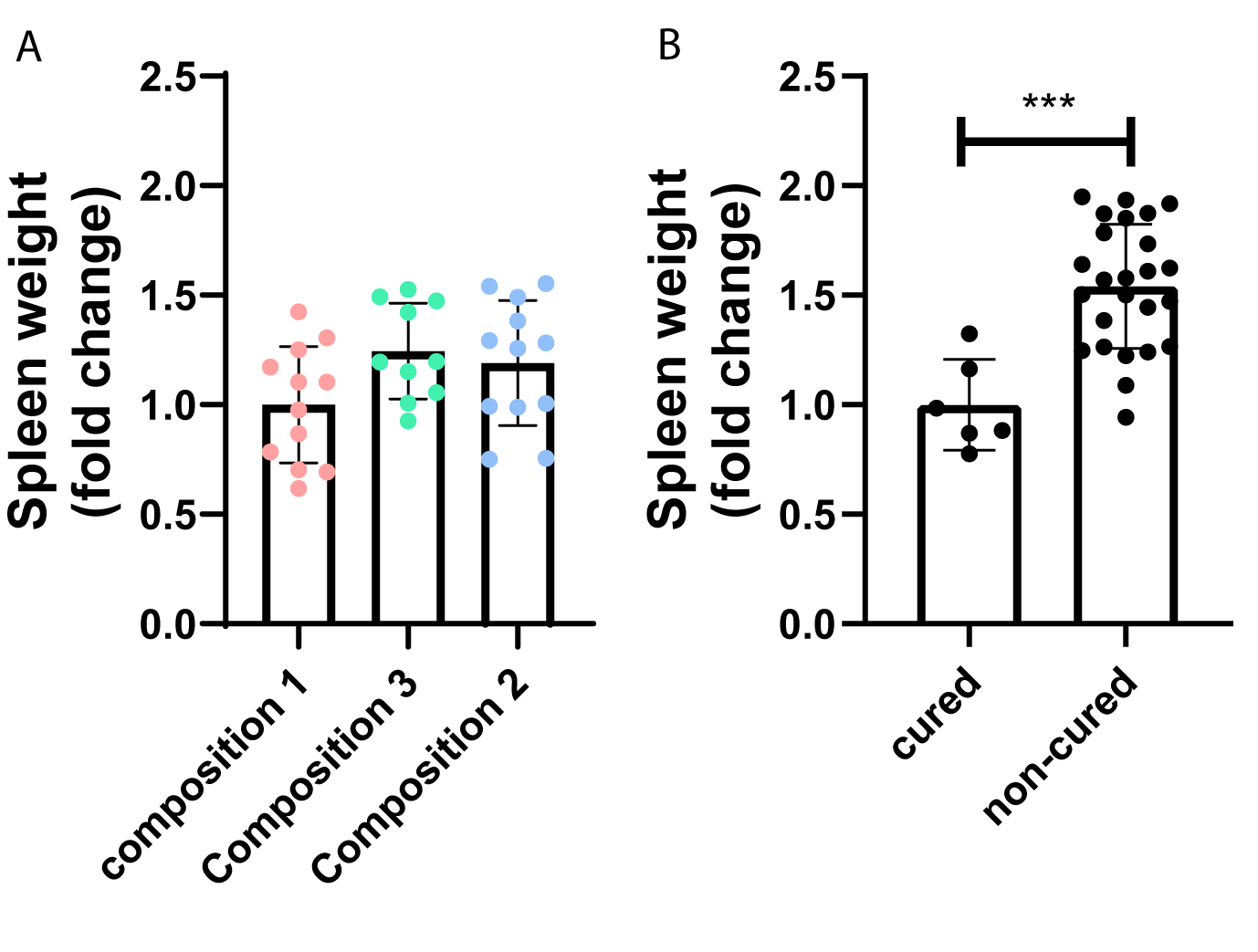

Supplement: Supplementary file 6 — Supporting File 6: mnfr70370‐sup‐0006‐FigureS6.tif. [file MNFR-70-e70370-s004.tif]
