# Supplementary material for: The Effect of Dietary Fiber Compositions on the Therapeutic Outcome of Combined Radio‐ and Immunotherapy in a Preclinical Cancer Model
Source: Mol Nutr Food Res. 2026 Jan 20;70(2):e70370. doi: 10.1002/mnfr.70370 (PMC12820406; doi:10.1002/mnfr.70370)
Supplement: Supplementary file 7 — Supporting File 7: mnfr70370‐sup‐0007‐SupMat‐Figure‐Legends.docx. [file MNFR-70-e70370-s003.docx]

**Supplementary figure 1. Flow cytometry gating strategy to investigate splenic T cell, MDSCs and NK cell subpopulations**

**Supplementary figure 2. Dietary interventions do not influence tumor take**

Calculated days for the tumor to reach a volume of 150, 180 and 200mm3 based on the exponential growth curves. Outliner in composition 1 does not change statistics.

**Supplementary figure 3.** **The model does not develop cachexia and dietary fiber composition does not modulate food intake, body weight, muscle weight or strength**

(A) Body weight curves from tumor cell injection (day -10 and -9) until sacrifice represented as mean ± SD per group. Day 0 indicates the day at which the tumor reached the starting volume of ± 200mm3. From day 30 onwards, SD becomes larges as animals are dropping out. (B) Average daily food intake calculated from tumor injection until sacrifice. (C) Comparisons of wet hind-leg muscle masses, weighted in pairs, from animals receiving compositions 1, 2 and 3. Wet muscle weight are corrected for body weight for each mouse individually. For statistical testing, the average muscle weight in composition 1 was determined and set to 1 and each mouse was normalized to this value individually. (D) Comparisons mean grip strengths over time from animals receiving compositions 1, 2 and 3.

**Supplementary figure 4. Analysis of effects gut microbiome composition changes compared to diet 2**

Heatmaps depicting the changes in genera comparing compositions 1 and 3 to composition 2, prior RT/IT (A), after RT/IT (B) and at sacrifice (C).

**Supplementary figure 5. Enrichment of** ***Atopobiaceae Family* and depletion of *Bacteroides* in fecal samples from cured animals**

Comparison of the relative abundance of *Atopobiaceae Family* and *Bacteroides* in fecal samples collected at sacrifice of the animals receiving composition 1.

**Supplementary figure 6. Cured mice had smaller spleens compared to non-cured mice**

At sacrifice, the spleen was isolated and weighted before processing into single cells. Spleen weights were corrected for body weight at sacrifice for each mouse individually. Comparisons of spleen weights between the diet compositions (A) and cured/non-cured mice (B). For statistical testing, the average spleen weight in composition 1, or cured, respectively, was determined and set to 1. Each mouse was normalized to this value individually.
