# Supplementary material for: The Effect of Dietary Fiber Compositions on the Therapeutic Outcome of Combined Radio‐ and Immunotherapy in a Preclinical Cancer Model
Source: Mol Nutr Food Res. 2026 Jan 20;70(2):e70370. doi: 10.1002/mnfr.70370 (PMC12820406; doi:10.1002/mnfr.70370)
Supplement: Supplementary file 8 — Supporting File 8: mnfr70370‐sup‐0008‐TableS1.docx. [file MNFR-70-e70370-s002.docx]

**Supplementary table 1: Composition of the fiber diets**

|  | **Composition 1 (g/kg)** | **Composition 2**  **(g/kg)** | **Composition 3**  **(g/kg)** |
| --- | --- | --- | --- |
| **Carbohydrates** | | | |
| **Cornstarch** | 452,9 | 452,9 | 452,9 |
| **Dextrinized cornstarch** | 151,3 | 151,3 | 151,3 |
| **Sucrose** | 97,3 | 97,3 | 97,3 |
| **Fiber** | | | |
| **Cellulose** | 47,5 | 0 | 0 |
| **Corn arabinoxylan** | 0 | 0 | 23,75 |
| **Rice arabinoxylan** | 0 | 11,875 | 0 |
| **Oat b-glucan** | 0 | 0 | 9,5 |
| **Low viscosity pectin** | 0 | 11,875 | 9,5 |
| **Resistant starch** | 0 | 0 | 4,75 |
| **Yeast b-glucan** | 0 | 11,875 | 0 |
| **Prebiotic oligosaccharides** | 0 | 11,875 | 0 |
| **Protein** | | | |
| **Casein** | 140 | 140 | 140 |
| **Fat** | | | |
| **Soybean oil** | 38,63 | 38,63 | 38,63 |
| **Others** | | | |
| **AIN93M mineral mix** | 35 | 35 | 35 |
| **AIN93M vitamin mix** | 10 | 10 | 10 |
| **Choline Bitratrate** | 2,5 | 2,5 | 2,5 |
| **tBHQ** | 0,008 | 0,008 | 0,008 |
| **Demi-water** | 24,6 | 24,6 | 24,6 |
| **Total sum** | 1000 | 1000 | 1000 |
| **All diets contain the same amount of calories (3821 Kcal)** | | | |

tBHQ: tert-butylhydroquinone
