# Supplementary material for: The Effect of Dietary Fiber Compositions on the Therapeutic Outcome of Combined Radio‐ and Immunotherapy in a Preclinical Cancer Model
Source: Mol Nutr Food Res. 2026 Jan 20;70(2):e70370. doi: 10.1002/mnfr.70370 (PMC12820406; doi:10.1002/mnfr.70370)
Supplement: Supplementary file 9 — Supporting File 9: mnfr70370‐sup‐0009‐TableS2.docx. [file MNFR-70-e70370-s009.docx]

**Supplementary table 2:** **Surface marker antibodies for flow cytometry on *ex vivo* splenocytes**

| **Marker** | **Fluorophore** | **Dilution** | **Product number, Brand** |
| --- | --- | --- | --- |
| Anti-CD45 | Bright Violet 421 (BV421) | 1/100 | 103133, Bio-Legend |
| Anti-CD3 | Allophycocyanin (APC) | 1/200 | 565643, BD Pharmingen™ |
| Anti-CD4 | Fluorescein isothiocyanate (FITC) | 1/100 | 11-0042-85, E Bioscience |
| Anti-CD8 | Peridinin-Chlorophyll-Protein (PerCP) | 1/100 | 553036, BD Pharmingen |
| Anti-CD44 | Allophycocyanin Cyanine 7 (APC-Cy7) | 1/100 | 560568, BD Bioscience |
| Anti-CD127 | Phycoerythrin (PE) | 1/100 | 552543, BD Bioscience |
| Anti-CD11b | Phycoerythrin Cyanine 7 (PE-Cy7) | 1/300 | 552850, BD Pharmingen |
| Anti-Gr-1 | Phycoerythrin (PE) | 1/300 | 553128, BD Bioscience |
| Anti-NK1.1 | Bright Violet 421 (BV421) | 1/100 | 562921, BD Horizon |
| **Antibodies for single staining controls** | | | |
| Anti-CD45 | Horizon V500 (V500) | 1/100 | 561487, BD Bioscience |
| Anti-CD45 | Bright Violet 421 (BV421) | 1/100 | 103133, Bio-Legend |
| Anti-CD3 | Allophycocyanin (APC) | 1/200 | 565643, BD Pharmingen™ |
| Anti-CD45 | Fluorescein isothiocyanate (FITC) | 1/100 | 553080, BD Bioscience |
| Anti-CD45 | Peridinin-Chlorophyll-Protein (PerCP) | 1/100 | 102129, Biolegend |
| Anti-CD45 | Allophycocyanin Cyanine 7 (APC-Cy7) | 1/100 | 103115, Biolegend |
| Anti-CD45 | Phycoerythrin (PE) | 1/100 | 553081, BD Bioscience |
| Anti-CD45 | Phycoerythrin Cyanine 7 (PE-Cy7) | 1/100 | 552848, BD Bioscience |
