# Supplementary material for: The Effect of Dietary Fiber Compositions on the Therapeutic Outcome of Combined Radio‐ and Immunotherapy in a Preclinical Cancer Model
Source: Mol Nutr Food Res. 2026 Jan 20;70(2):e70370. doi: 10.1002/mnfr.70370 (PMC12820406; doi:10.1002/mnfr.70370)
Supplement: Supplementary file 10 — Supporting File 10: mnfr70370‐sup‐0010‐TableS3.docx. [file MNFR-70-e70370-s007.docx]

**Supplementary table 3. Permanova analysis comparing the microbiota community structure (between-sample Aitchison distance) in the fecal samples.**

| **Time-point** | **Comparison** | **Variable** | **Df** | **SumOfSqs** | **R^2^** | **F** | **Pvalue** | **PAdjusted** |
| --- | --- | --- | --- | --- | --- | --- | --- | --- |
| pre-treatment | Composition 1 versus Composition 3 | outcome | 1 | 4264,861 | 0,036 | 0,983 | 0,386 | 0,534 |
|  |  | diet | 1 | 30162,414 | 0,254 | 6,955 | 0,001 | 0,002 |
|  |  | Residual | 19 | 82400,620 | 0,694 |  |  |  |
|  |  | Total | 21 | 118773,095 | 1,000 |  |  |  |
| pre-treatment | Composition 1 versus Composition 2 | outcome | 1 | 3623,370 | 0,030 | 0,818 | 0,671 | 0,755 |
|  |  | diet | 1 | 26833,696 | 0,221 | 6,056 | 0,001 | 0,002 |
|  |  | Residual | 20 | 88615,145 | 0,731 |  |  |  |
|  |  | Total | 22 | 121263,334 | 1,000 |  |  |  |
| pre-treatment | Composition 3 versus Composition 2 | outcome | 1 | 3224,880 | 0,039 | 0,879 | 0,639 | 0,755 |
|  |  | diet | 1 | 12944,199 | 0,157 | 3,529 | 0,001 | 0,002 |
|  |  | Residual | 18 | 66014,713 | 0,803 |  |  |  |
|  |  | Total | 20 | 82237,810 | 1,000 |  |  |  |
| post-treatment | Composition 1 versus Composition 3 | outcome | 1 | 3446,986 | 0,028 | 0,752 | 0,713 | 0,755 |
|  |  | diet | 1 | 31339,915 | 0,253 | 6,833 | 0,001 | 0,002 |
|  |  | Residual | 19 | 87142,880 | 0,704 |  |  |  |
|  |  | Total | 21 | 123827,157 | 1,000 |  |  |  |
| post-treatment | Composition 1 versus Composition 2 | outcome | 1 | 3638,730 | 0,026 | 0,691 | 0,884 | 0,884 |
|  |  | diet | 1 | 28543,165 | 0,205 | 5,421 | 0,001 | 0,002 |
|  |  | Residual | 20 | 105302,549 | 0,758 |  |  |  |
|  |  | Total | 22 | 138996,246 | 1 |  |  |  |
| post-treatment | Composition 3 versus Composition 2 | outcome | 1 | 4158,085 | 0,039 | 0,890 | 0,601 | 0,755 |
|  |  | diet | 1 | 18482,222 | 0,173 | 3,958 | 0,001 | 0,002 |
|  |  | Residual | 18 | 84051,315 | 0,788 |  |  |  |
|  |  | Total | 20 | 106728,367 | 1,000 |  |  |  |
| endpoint | Composition 1 versus Composition 3 | outcome | 1 | 5340,348 | 0,045 | 1,191 | 0,202 | 0,303 |
|  |  | diet | 1 | 27217,858 | 0,227 | 6,071 | 0,001 | 0,002 |
|  |  | Residual | 19 | 85178,424 | 0,710 |  |  |  |
|  |  | Total | 21 | 119929,317 | 1,000 |  |  |  |
| endpoint | Composition 1 versus Composition 2 | outcome | 1 | 7309,653 | 0,055 | 1,513 | 0,07 | 0,126 |
|  |  | diet | 1 | 25748,459 | 0,192 | 5,330 | 0,001 | 0,002 |
|  |  | Residual | 20 | 96608,981 | 0,721 |  |  |  |
|  |  | Total | 22 | 133943,072 | 1,000 |  |  |  |
| endpoint | Composition 3 versus Composition 2 | outcome | 1 | 5421,069 | 0,051 | 1,152 | 0,194 | 0,303 |
|  |  | diet | 1 | 15144,101 | 0,144 | 3,218 | 0,002 | 0,004 |
|  |  | Residual | 18 | 84700,510 | 0,804 |  |  |  |
|  |  | Total | 20 | 105406,372 | 1,000 |  |  |  |

Statistical analysis was 2-sided and p-values were corrected for multiple testing using Benjamini-Hochberg method. P values smaller than 0.05 were considered statistically significant. Df, degrees of freedom, SumOfSqs, sum of squares.
